# Understanding the Role of Silver Nanostructures and Graphene Oxide Applied as Surface Modification of TiO_2_ in Photocatalytic Transformations of Rhodamine B under UV and Vis Irradiation

**DOI:** 10.3390/ma13204653

**Published:** 2020-10-19

**Authors:** Kaja Spilarewicz-Stanek, Anna Jakimińska, Aneta Kisielewska, Damian Batory, Ireneusz Piwoński

**Affiliations:** 1 Department of Materials Technology and Chemistry, Faculty of Chemistry, University of Lodz, Pomorska 163, 90-236 Lodz, Poland; spilarewicz.stanek@gmail.com (K.S.-S.); aneta.kisielewska@chemia.uni.lodz.pl (A.K.); 2 Faculty of Chemistry, Jagiellonian University, Gronostajowa 2, 30-387 Krakow, Poland; anna.jakiminska@doctoral.uj.edu.pl; 3 Department of Vehicles and Fundamentals in Machine Design, Lodz University of Technology, Stefanowskiego 1/15, 90-924 Lodz, Poland; damian.batory@p.lodz.pl

**Keywords:** TiO_2_, Rhodamine B, Rhodamine 110, photocatalysis, surface architecture

## Abstract

This work is focused on photocatalytic properties of titanium dioxide thin coatings modified with silver nanostructures (AgNSs) and graphene oxide (GO) sheets which were analyzed in processes of chemical transformations of rhodamine B (RhB) under ultraviolet (UV) or visible light (Vis) irradiation, respectively. UV-Vis spectroscopy was applied to analyze the changes in the RhB spectrum during photocatalytic processes, revealing decolorization of RhB solution under UV irradiation while the same process coexisting with the transformation of RhB to rhodamine 110 was observed under Vis irradiation. The novelty of this study is the elaboration of a methodology for determining the parameters characterizing the processes occurring under the Vis irradiation, which enables the comparison of photocatalysts’ activity. For the first time, the method for quantification of rhodamine B transformation into rhodamine 110 in the presence of a semiconductor under visible light irradiation was proposed. Photocatalysts with various surface architectures were designed. TiO_2_ thin coatings were obtained by the sol-gel method. GO sheets were deposited on their surface using the dip-coating method. AgNSs were photogenerated on TiO_2_ or grown spontaneously on GO flakes. For characterization of obtained photocatalysts, scanning electron microscopy (SEM), X-ray diffraction (XRD) and diffuse-reflectance spectroscopy (DRS) techniques were applied. The results indicate that the surface architecture of prepared coatings does not affect the main reaction path but have an influence on the reaction rates and yields of observed processes.

## 1. Introduction

The design of the architecture of photocatalysts’ surfaces in nanoscale is a promising approach to photocatalysis, which enables detailed comprehension of matter behavior [1]. It is known, that the improvement of photocatalytic activity of semiconductors, can be achieved by inter alia defined porosity [2,3], exposition of the selected crystallographic facets [4,5,6], hierarchical structure [7,8,9], decoration with nanoparticles [10,11] and quantum dot incorporation [12,13,14], which enables the limitations of conventional photocatalysts to be overcome. It is believed that the development of multi-component systems can be an effective method for obtaining excellent photocatalysts due to the introduction of complementary coexisting physicochemical phenomena including, e.g., better light-harvesting, photosensitization, efficient electron-hole separation or tailoring the band-gap of the semiconductors [15]. This strategy is very promising in terms of achieving high photocatalytic processes efficiency and the possibility of conducting these processes under solar light illumination.

Ternary hybrid nanocomposites based on TiO_2_ combined with 2D materials and metal nanoparticles, such as graphene oxide (GO) and silver nanoparticles (AgNSs), provide new insight into photocatalytic studies. Both components introduced to TiO_2_ possess the ability to enhance light absorption and reduce the charge carriers’ recombination [16,17]. The incorporation of GO also leads to intensified adsorption of organic compounds [17]. Therefore the combination of those components with TiO_2_ can be advantageous. However, the control of photocatalyst morphology is also highly recommended to obtain desirable properties. For example, extensive surface coverage with silver nanoparticles can negatively affect the photocatalytic activity of decorated semiconductor, due to the possibility of nanoparticles acting as the recombination centers [18,19]. Furthermore, excess of reduced graphene oxide loaded in composite with TiO_2_ can limit photocatalyst activity through the occurrence of a shielding effect [20]. Some reports indicate that GO under photocatalytic process conditions undergoes reduction and degradation [21,22]. Mentioned phenomena can also affect multi-component systems efficiency. Therefore, extensive research on surface architecture should be performed in order to analyze the mutual dependence of photocatalytic activity.

Several reports focused on studies of TiO_2_ combined with GO flakes and AgNSs have been presented. Sim et al. [23] demonstrated effective degradation of methylene blue and 2-chlorophenol using TiO_2_ nanotube arrays with deposited GO flakes and AgNSs under visible light (Vis) irradiation. The ternary system exhibited an improvement of photocatalytic activity in comparison with binary photocatalysts. Similar observations were reported by Liu et al. [24], who proposed the method to obtain GO flakes decorated with TiO_2_ nanorods and AgNSs. Results presented in this work shows that the content of components moderately influences the photocatalytic activity of ternary systems, which was estimated by photodegradation studies of phenol and acid orange 7 under solar irradiation. The analysis of the impact of the photocatalyst composition on photocatalytic activity was also discussed in the work of Qi et al. [25]. They demonstrated that GO flakes with deposited TiO_2_ mesocrystals and AgNSs were effective in the decomposition of Rhodamine B and dinitro butyl-phenol under Vis illumination. However, photocatalytic studies reveal that high AgNSs content in nanocomposite contributes attenuation of the ternary photocatalyst activity. The reports mentioned above explain the increase of photocatalytic activity of TiO_2_ by the synergistic effect of components cooperation in ternary systems. However, Alsharaed et al. [26] described how Ag/TiO_2_-GO nanohybrid revealed enhancement in photocatalytic decomposition of phenol only under Vis irradiation. By contrast, upon ultraviolet (UV) irradiation TiO_2_ modified only by AgNSs was more active than a ternary photocatalyst. Consequently, the cooperation of components in composite photocatalysts needs further elucidation.

In particular, the impact of photocatalyst composition on the behavior of dyes under a selected range of radiation is interesting. Chromophore in the chemical structure of xanthene dyes enables excitation of the molecule under Vis illumination [27,28]. This phenomenon can be used for the sensitization of TiO_2_, which itself cannot be excited with this range of light. Therefore, the photocatalytic reaction paths for dyes transformations under UV and Vis light irradiation can have different mechanisms. In this study, rhodamine B, as a commonly studied model organic pollutant [29], was selected for the elucidation of these phenomena in ternary photocatalytic systems based on TiO_2_, GO, and AgNSs. The novelty of the present paper is an investigation of three component systems consisting of TiO_2_, GO and AgNSs and how the assembly of these constituents in one coating affect the photocatalytic performance. Three types of the architecture of the ternary coatings were designed and successfully prepared for the purpose of analyzing the impact of photocatalysts’ morphology under selected irradiation range: UV and Vis. The significance of the present work is to show that the appropriate combination of catalyst components can have a substantial influence on its performance. The novel methodology for quantification of rhodamine B transformation into rhodamine 110 was proposed. Also, processes of rhodamine B decomposition under both ranges of radiation were discussed.

## 2. Experimental

### 2.1. Materials

Silicon wafers exposing the (100) surface (ITME—Institute of Electronic Materials Technology, Warsaw, Poland) were selected as a substrates for TiO_2_ thin coatings preparation. Reagents for sol preparation: titanium tetraisopropoxide (Aldrich, 99.7%), isopropanol (Avantor Performance Materials Poland S.A., pure, min. 99.7%) and hydrochloric acid (Chempur, 11 M, pure for analysis) diluted to concentration of 2 M were used without further purification. Graphene oxide (GO) was used as a water dispersion at the concentration of 2 mg/mL (Sigma-Aldrich, St. Louis, MO, USA). Silver nitrate (Avantor Performance Materials Poland S.A., 99.85%) solution at the concentration of 0.1 mM was prepared in ethanol (Avantor Performance Materials Poland S.A., pure, min. 99.7%). Rhodamine B (Sigma Aldrich, pure ~95%) solution at the concentration of 1 × 10^−5^ M was prepared in deionized water. Deionized water was obtained by purification using Millipore Simplicity UV system (18.2 MΩ cm at 25 °C, New York, NY, USA).

### 2.2. Preparation of Titanium Dioxide Coatings

Firstly, the silicon substrates were cleaned in a mixture of ethanol and isopropanol in an ultrasonic bath, then with dust-free cloth moistened with ethanol and lastly a stream of compressed air. Titanium dioxide coatings were obtained using the sol-gel method, reported in our previous studies [30,31]. Briefly, titanium tetraisopropoxide and isopropanol with the addition of HCl as a catalyst were stirred for 20 min. Furthermore, prepared sol was applied on silicon wafers substrates using the dip-coating method. Each of the obtained photocatalysts had the same surface area equal to 1 cm^2^. Dipping at the immersion-withdrawal velocity of 25 mm/min was repeated three times with preserving a drying time of 15 min between repetitions. Obtained coatings were annealed at 100 °C and calcined at 500 °C (both for 2 h), leading to obtain anatase phase.

### 2.3. Surface Modification of TiO_2_ Coatings with Graphene Oxide (GO) and Silver Nanostructures (AgNSs)

**Figure 1:**
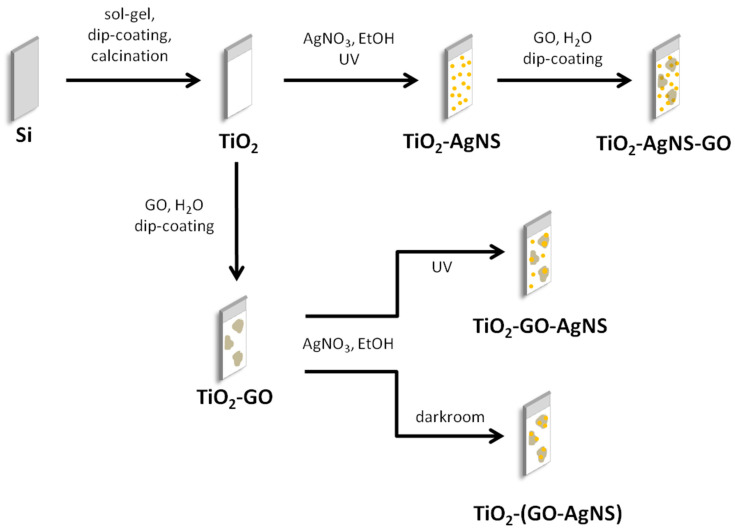
The schematic illustration of the preparation steps of photocatalysts with different surface architecture.

Figure 1 shows a scheme illustrating preparation of two-component and three-component photocatalysts. GO water dispersion in concentration of 0.5 mg/mL was obtained by dilution of stock GO dispersion (2 mg/mL) with deionized water and then, it was used for surface modification of selected coatings (TiO_2_ or TiO_2_-AgNSs). GO flakes were transferred onto substrates using dip-coating technique at the immersion-withdrawal velocity of 25 mm/min.

Silver nanostructures (AgNSs) on the photocatalysts surfaces were obtained by photoreduction of Ag^+^ ions. Therefore, AgNO_3_ ethanol solution at the concentration of 0.1 mM was used as a precursor. Previously prepared coatings (TiO_2_ or TiO_2_-GO) were immersed in polymethacrylate (PMMA) cuvettes containing 3.5 mL of AgNO_3_ ethanol solution for 5 min, and during this time cuvettes were irradiated with a UV lamp (Consulting Peschl, 2 × 15 W, wavelength λ_max_ = 365 nm, 15 mW/cm^2^). According to the supplier’s specification, selected cuvettes can be applied in the wavelength range from 220 to 900 nm and are transparent at λ_max_ = 365 nm. Subsequently, the coatings were rinsed with deionized water to remove residual ions/solution. On the contrary, the selective decoration of GO sheets deposited on TiO_2_ coatings with AgNSs was performed without introducing light, as in method reported previously [32]. Briefly, coatings were immersed in cuvettes with 3.5 mL of AgNO_3_ ethanol solution and left in a darkroom for 5 min. After this time coatings also were rinsed with water, as in the case of photoreduction.

### 2.4. Characterization

Morphology of prepared photocatalysts was analyzed using a field-emission scanning electron microscope (FE-SEM—FEI NovaNano SEM 450, FEI, Hillsboro, OR, USA) equipped with a Schottky gun. Images were obtained with the use of the Everhart–Thornley detector (ETD, FEI, Hillsboro, OR, USA) or the through lens detector (TLD, FEI, Hillsboro, OR, USA) in immersion mode. Analysis of surface coverage by GO and AgNSs was estimated using Image J 1.52a software [33].

The diffraction spectra were recorded using an Empyrean PANalytical X-ray diffractometer (XRD, Almelo, Netherlands) equipped with a Co lamp (wavelength λ = 0.1790 nm). Signals were collected in the range of 2θ = 20–120°, using the angle of incidence 0.5° in 0.1° steps. The counting time was 20 sec per step. 2θ_Co_ was scaled to the values corresponding to 2θ_Cu_ using the Rietveld equation in order to compare with the values given in the literature.

Diffuse reflectance spectra were recorded with a UV/Vis diffuse-reflectance spectroscopy (DRS) spectrophotometer PerkinElmer Lambda 25 (Waltham, MA, USA), and band gap energy was determined with the use of Kubelka–Munk equation and Tauc method.

### 2.5. Analysis of Photocatalytic Activity

The photocatalysts obtained were immersed in quartz cuvettes filled with 2.5 mL of rhodamine B (RhB) water solution with a concentration of 1 × 10^−5^ M. Before irradiation cuvettes were placed in a dark room for 20 min, receiving a adsorption-desorption equilibrium. During the experiment, cuvettes with air access were placed in front of a xenon lamp (150 W, Instytut Fotonowy, Kraków, Poland) equipped with a UV or Vis cut-off filter, at a distance of 15 cm and 25 cm, respectively. Intensity of radiation was equal to 223 mW/cm^2^ in both cases. Changes in RhB spectrum and concentration were monitored using UV-Vis spectrophotometer (USB2000+, Ocean Optics, Dunedin, FL, USA).

## 3. Results and Discussion

### 3.1. Titanium Dioxide Coatings Characterization

**Figure 2:**
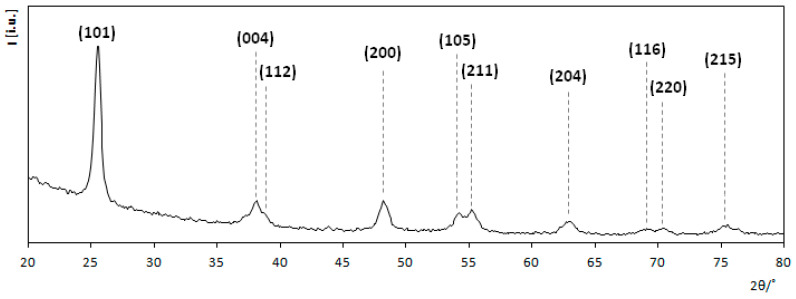
X-ray diffraction pattern of bare TiO_2_ coating.

Initially, the titanium dioxide coating was prepared using the sol-gel method. In order to obtain TiO_2_ in anatase form the calcination process was performed at 500 °C. The crystallographic structure of the coating was verified using X-ray diffraction (XRD). Figure 2 represents a converted diffraction pattern with Miller indices assigned to characteristic anatase peaks. The most characteristic peaks for anatase occur at 2θ values equal to 25.3°, 37.8°, 48.1°, 53.9° and 55.1° which correspond to the crystallographic planes: (101), (004), (200), (105) and (211) respectively [34,35]. Since there are no signals in the obtained spectrum, indicating the presence of other crystalline phases, it can be stated that the obtained TiO_2_ coating is pure anatase.

**Figure 3:**
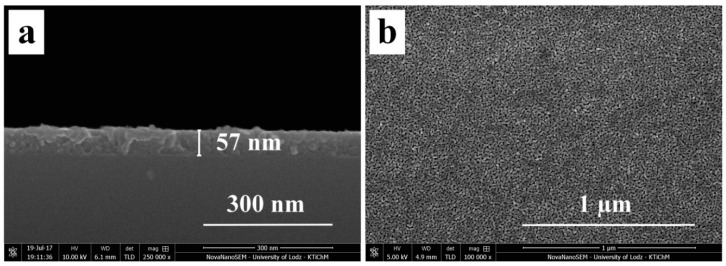
Scanning electron microscopy (SEM) characterization of bare TiO_2_ coating: (**a**) cross-view, (**b**) top-view.

For further characterization of the obtained coating, scanning electron microscopy (SEM) was undertaken. Figure 3a represents a cross-section of the TiO_2_ layer deposited on the silicon wafer. The average coating thickness is approximately 57 ± 3 nm. The layer is uniformly thick over the entire length. Figure 3b presents the SEM image of the coating surface, which indicates the occurrence of regular porosity with pore sizes not exceeding 2 nm, visible as darker fields. Porosity is the result of the formation of anatase crystal phase during the calcination process, which is distinctive of thin coatings obtained by the sol-gel method [35].

### 3.2. Surface Modifications with AgNSs and GO

**Figure 4:**
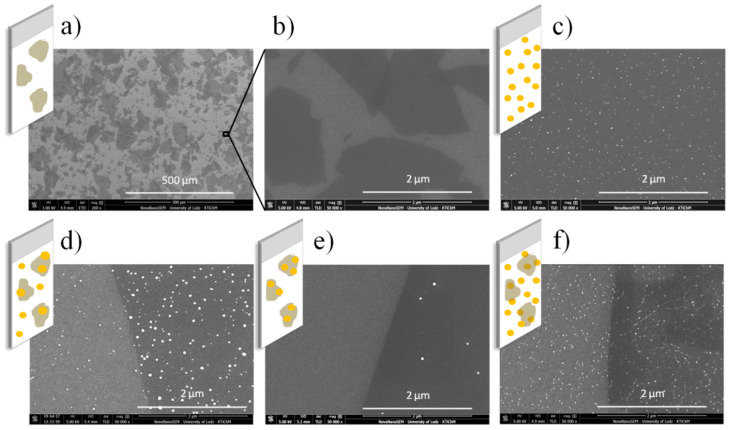
SEM images and related schemes of (**a**) and (**b**) TiO_2_-GO (graphene oxide), (**c**) TiO_2_-AgNSs (silver nanostructures), (**d**) TiO_2_-GO-AgNSs, (**e**) TiO_2_-(GO-AgNSs) and (**f**) TiO_2_-AgNSs-GO.

In order to analyze the impact of photocatalysts architectures on their photocatalytic activity, different TiO_2_ surface modifications by GO and AgNSs were obtained. Figure 4 depicts SEM images of prepared photocatalysts and related schemes: two-component coatings: TiO_2_-GO, TiO_2_-AgNSs and three-component coatings: TiO_2_-GO-AgNSs, TiO_2_-(GO-AgNSs) and TiO_2_-AgNSs-GO. Explanation of schemes: white area—TiO_2_ thin film, yellow dots—AgNSs, grey objects—GO flakes.

Firstly the TiO_2_ coatings were used as the substrates for GO deposition. GO sheets were applied on the surface of the photocatalysts using the dip-coating method as described in the experimental section. Figure 4a depict randomly distributed GO flakes on the coating surface, visible as dark objects. Flakes reveal the slight presence of wrinkles, which is characteristic of explicit adhesion to the surface. It was found that GO flakes occupy approximately 50% of the surface of the photocatalyst.

For the purpose of decoration of TiO_2_ surface with AgNSs, photocatalytic properties of TiO_2_ were used. During UV irradiation, electrons from the valence band (VB) of TiO_2_ were transferred to the conduction band (CB), leading to the formation of an electron-hole pair, according to the reaction equation:

TiO_2_ + *hv* → TiO_2_ (e^−^ + h^+^) (1)

Photogenerated electrons reduce silver ions adsorbed on TiO_2_ surface, resulting in silver nanostructures’ growth [36]:

TiO_2_ (e^−^) + Ag^+^ → TiO_2_ + Ag^0^ (2)

At the same time, ethanol acts as a hole scavenger improving the efficiency of photocatalytic growth of AgNSs [37,38]:

TiO_2_ (h^+^) + C_2_H_5_OH → TiO_2_ + ^•^C_2_H_5_OH (3)

The SEM image presented in Figure 4c shows representative morphology of obtained TiO_2_-AgNSs photocatalyst. It can be seen that AgNSs grown on TiO_2_, visible as white objects, are single-entity and quasi-spherical. The average estimated size of AgNSs was 18 ± 3 nm, and the maximal diameter did not exceed 37 nm. The surface density of obtained AgNSs equals 55 AgNSs/µm^2^, and they occupy 1.1% of TiO_2_ surface.

In the last step TiO_2_ coatings modified with both GO and AgNSs were received. Since it was assumed that the way of combining the components could have a significant impact on the photocatalytic properties of the composites, three types of photocatalysts were prepared: TiO_2_-AgNSs-GO, TiO_2_-GO-AgNSs and TiO_2_-(GO-AgNSs). To obtain TiO_2_-AgNSs-GO composite, a previously obtained TiO_2_-AgNSs photocatalyst was used as a substrate for dip-coating deposition of GO flakes. Whereas previously received TiO_2_-GO coatings were used for preparation of TiO_2_-GO-AgNSs and TiO_2_-(GO-AgNSs). In the first case TiO_2_-GO was immersed in AgNO_3_ ethanol solution and irradiated with UV light at the same conditions as in preparation of TiO_2_-AgNSs mentioned above, resulting in coverage of TiO_2_ and GO by AgNSs (Figure 4d). By contrast, for the purpose of TiO_2_-(GO-AgNSs) preparation, growth of AgNSs merely on GO flakes deposited on TiO_2_ was performed without exposure to light (Figure 4e).

SEM images of TiO_2_-AgNSs-GO, TiO_2_-GO-AgNSs and TiO_2_-(GO-AgNSs) photocatalysts presented in Figure 4d–f, confirm that these coatings differ in the distribution of AgNSs (Table 1), which is the result of the preparation method. In the case of TiO_2_-GO-AgNSs surface, AgNSs are visible on both uncovered TiO_2_ and covered by GO. Here, the growth of AgNSs was stimulated by photogenerated electrons from the excited TiO_2_ surface. By contrast, AgNSs were grown only on the GO sheets on TiO_2_-(GO-AgNSs) surface. Growth of AgNSs on GO was possible due to chemical properties of this material [32,39,40]. In our previous study [32] it was proved that the presence of oxygen functional groups on the graphene oxide surface provides reactive sites for the spontaneous chemical reduction of Ag^+^ ions, leading to formation of AgNSs. It was found that hydroxyl groups were mainly responsible for AgNSs’ nucleation. These results were observed after immersion of GO deposited on Si substrate in AgNO_3_ ethanol solution carried out in a darkroom. The conditions of this experiment were consistent with those in the present work.

Taking a closer look at the characteristic parameters of AgNSs, certain differences can be observed comparing the results obtained for Si-GO-AgNSs [32] with TiO_2_-(GO-AgNSs). The AgNSs size (3 ± 2 nm, max 20 nm) found on Si-(GO-AgNSs) is smaller than that noted on TiO_2_-(GO-AgNSs)—average size 19 ± 3 nm, max 53 nm. Simultaneously, surface density for Si-(GO-AgNSs) which equals to 203 AgNSs/µm^2^ is definitely larger than that for TiO_2_-(GO-AgNSs)—3 AgNSs/µm^2^. The reason for these differences is related to the nature of the applied substrate. Presumably, hydroxyl groups from GO interact more intensely with hydroxyl groups localized on TiO_2_ surface than on Si. Consequently, most of the GO hydroxyl groups are involved in creating interactions with the TiO_2_ surface. This results in fewer free functional groups, providing less nucleation sites for AgNSs growth, which translates further into their larger sizes.

**Figure 5:**
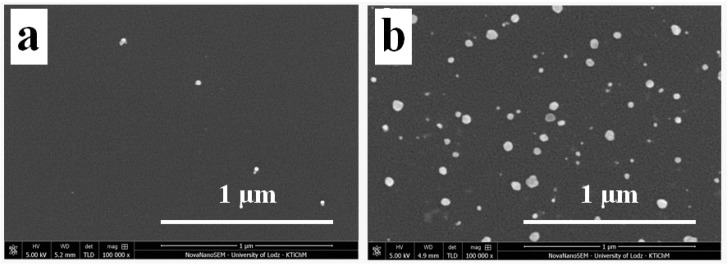
The morphology of GO area on TiO_2_-(GO-AgNS) (**a**) and on TiO_2_-GO-AgNS (**b**) coatings at a magnification of 100,000 ×.

**Figure 6:**
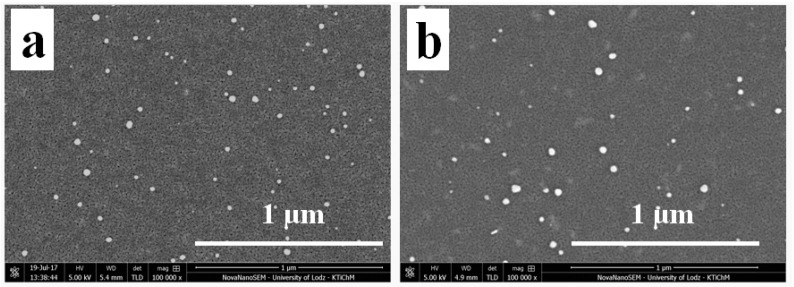
The morphology of TiO_2_-AgNS (**a**) and the area of TiO_2_ in the TiO_2_-GO-AgNS coating (**b**) at a magnification of 100,000 ×.

When the generation of AgNSs is performed under UV irradiation, the surface decoration turns out different. Analyzing TiO_2_-GO-AgNSs surface coverage, where the AgNSs growth was stimulated by the participation of UV irradiation, almost 12 times more AgNSs (35 AgNSs/µm^2^) were formed on GO flakes than on flakes deposited on the TiO_2_-(GO-AgNSs), where the modification was carried out without an access of light (Figure 5). At the same time as the AgNSs number increases, the resulting nanostructures are also characterized by larger sizes—the maximum size increased from 53 nm to 66 nm, and the average size from 19 ± 3 nm to 26 nm ± 4. This is clear evidence that the growth process was stimulated by another factor. Lightcap and co-authors [38] proved that titanium dioxide excited by UV irradiation transfers an electron to GO, which then migrates inside the flake to adsorbed Ag^+^ ions on its surface, resulting in their reduction to metallic silver. On this basis, it can be concluded that electrons photoinduced in TiO_2_ are involved in AgNSs growth on the GO deposited on the TiO_2_ surface. Furthermore, this theory can be supported by the results of AgNSs photogeneration on the uncovered TiO_2_ surface in TiO_2_-GO-AgNSs coating in comparison with TiO_2_-AgNSs (Figure 6). The size of obtained AgNSs on both surfaces are similar, but the AgNSs surface density on the TiO_2_ area covered by GO in TiO_2_-GO-AgNSs is almost 2.75 times smaller. This phenomenon can be elucidated by the possibility that photogenerated electrons in TiO_2_-GO, which could take part in reduction of Ag^+^ on TiO_2_, are trapped in GO resulting in reduction of AgNSs surface density on the TiO_2_ surface in TiO_2_-GO-AgNSs composite compared to TiO_2_-AgNSs.

Moreover, an order of component application have an impact on an arrangement of flakes on the surface. In the case of TiO_2_-GO-AgNSs and TiO_2_-(GO-AgNSs), GO flakes lie flat on the surface of TiO_2_, and the AgNSs that are formed on them do not change the arrangement of flakes and their adhesion to the substrate. These flakes show only local multiplication of the GO layer as in the TiO_2_-GO coating described earlier. By contrast, in the case of TiO_2_-AgNSs-GO, where GO was deposited on previously grown silver nanostructures, small wrinkles are visible (in the form of characteristic white “lines” between AgNSs). Presence of AgNSs under GO flakes effects in surface unevenness, which increase the tendency of GO to wrinkling [41]. The arrangement of GO flakes can produce two effects. On the one hand any type of deformation in the structure of graphene materials has a negative impact on electrons’ movement in its structure. While on the other hand, the appearance of wrinkles allows the solution to penetrate between the surface of the photocatalyst and the GO flake, which can be advantageous from the viewpoint of photocatalysis.

**Figure 7:**
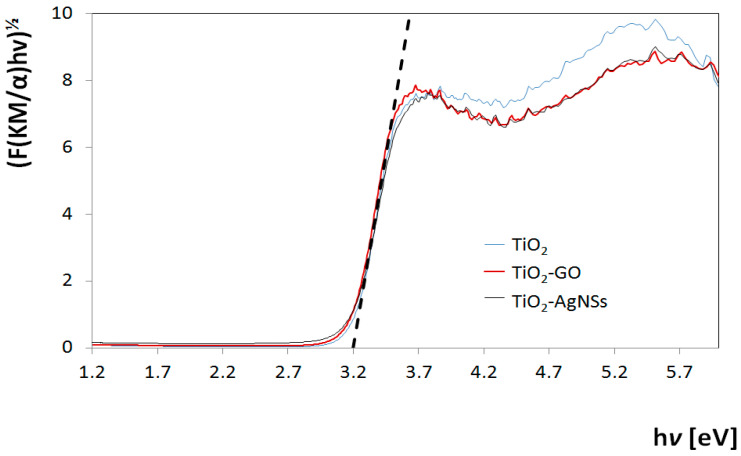
Tauc plot for TiO_2_ (blue line), TiO_2_-GO (red line), TiO_2_-GO-AgNSs (dark grey line).

A further characteristic of photocatalytic systems was performed by diffuse-reflectance spectroscopy (DRS). Figure 7 shows a graph which depicts the dependence of the Tauc function on the photon energy (F(KM/α)hν)½ = f(hν) for TiO_2_, TiO_2_-GO and TiO_2_-AgNSs coatings. The determined value of bandgap energy (E_g_) for these coatings equals 3.2 eV, which is typical of the anatase form of TiO_2_ [19,42]. The presence of AgNSs and GO on the TiO_2_ surface did not change the value of the semiconductor E_g_. The plot obtained for TiO_2_-AgNSs shows that localized surface plasmon resonance (LSPR) is not observed. This means that all changes in photocatalytic properties of obtained coatings, gained after modification with AgNSs and GO, were not caused by differences in E_g_ value.

### 3.3. Photocatalytic Transformations of Rhodamine B

The evaluation of photocatalytic properties of the obtained photocatalysts was performed by the spectrophotometric observation of changes in the RhB solution spectrum during irradiation. Experiments were carried out with aqueous solution of RhB in two ranges of light: UV and Vis. Two different phenomena were studied—a direct activation of photocatalyst and sensitization. On the one hand, UV light activate TiO_2_, providing photogeneration of hydroxyl radicals (^•^OH) and other reactive forms of oxygen. These species react with the RhB molecules leading to their decomposition [43,44]. On the other hand, Vis light does not excite TiO_2_, but can affect the RhB molecules leading to their excitation. Then, oxygen radicals arise on TiO_2_ by indirect sensitization process, after the transfer of electrons from lowest unoccupied molecular orbital (LUMO) of RhB molecules in excited state to the conduction band of TiO_2_ [45]. This process is possible due to the fact, that the LUMO energy level of rhodamine B is higher (−2.73 eV) [45] than the lower edge of the anatase conduction band (−4.0 to −4.3 eV) [46], and is widely applied in the field of dye-sensitized solar cells (DSSC’s) and also in photocatalysis [47,48].

**Figure 8:**
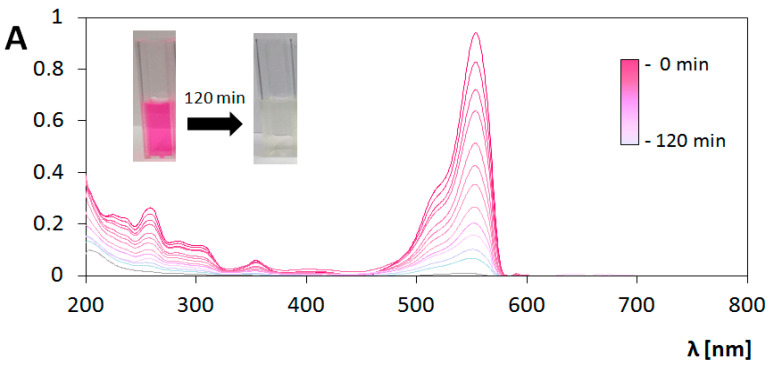
Changes in absorption spectrum of rhodamine B (RhB) solution during photocatalysis on bare TiO_2_ coating under ultraviolet (UV) illumination. Insert: change in color of solution.

**Figure 9:**
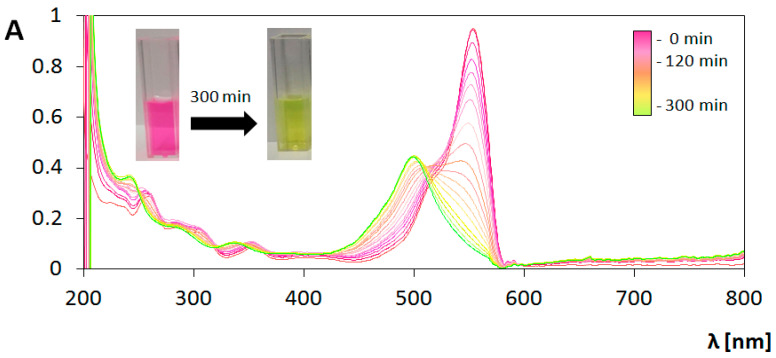
Changes in absorption spectrum of RhB solution during photocatalysis on bare TiO_2_ coating under visible light (Vis) irradiation. Insert: change in color of solution.

These two mechanisms of excitation result in different pathways of RhB decomposition. Figure 8 and Figure 9 contain compiled graphs, presenting the changes in absorption spectrum of RhB under UV and Vis light irradiation in the presence of bare TiO_2_ coating, respectively. In the case of UV irradiation, a decrease in the height of peak around 554 nm is observed without any significant changes in peaks positions, which is typical phenomenon for chromophore destruction in the process of RhB photodegradation. Measurement of absorbance value at the wavelength 554 nm were used for determination of reaction rate constant (k’) based on Langmuir–Hinshelwood’s model following the pseudo-first-order kinetics expressed as [49]:

(4) $$ln\frac{C}{C_{0}}\;=\;-\;k^{\prime }t$$

where: k’ is the pseudo-first-order rate constant (min^−1^), C_0_ the initial concentration of RhB and C is the concentration of RhB at the time *t*. This methodology is often used in the case of estimation of photocatalyst activity in dye photodecomposition [25,50,51,52,53,54].

However, for the determination of photocatalyst activity under Vis light irradiation, reaction rate constant parameter is not sufficient. Analysis of the graph presented in Figure 9 indicates two processes occurring simultaneously. During the elongation of exposure to Vis light, the initial absorbance value at the maximum of peak at the wavelength 554 nm decrease. At the same time the hypsochromic shift can be seen, and after 280 min of the photocatalytic process the peak with the maximum at 498 nm is extracted. After 280 min, the spectrum does not undergoes any changes. The peak with maximum at 498 nm was identified as a signal from rhodamine 110 (Rh-110) which is similar to results obtained in studies of photocatalytic transformation of rhodamine B in the presence of TiO_2_ [55,56,57], TiO_2_-Ag [55], TiO_2_-Si [57], CdS [55,58], Pb_3_Nb_4_O_13_ [59] irradiated by Vis light. The same effects were observed during photocatalytic measurements performed under Vis irradiation for every prepared type of coating analyzed in this study.

The process of photocatalytic transformation of RhB into Rh-110 via the N-deethylation mechanism was firstly described by Watanabe et al. [58]. In this process, ethyl groups are eliminated from the aminodiethyl groups of RhB, resulting in consecutively formation of N,N,N’-triethyl-Rh-110, N,N’-diethyl-Rh-110, N-ethyl-Rh-110 and Rh-110. These compounds have absorption maxima at the wavelength of 539 nm, 522 nm, 510 nm and 498 nm, respectively. Ethyl groups in RhB molecules structure act as the auxochromes, determining the location of the absorption maximum. Their elimination cause the hypsochromic shift shown in the spectrum. It was found that during elimination of ethyl groups from the adsorbed dye on photocatalyst surface, the excess negative charge accumulated on TiO_2_ is removed by the adsorbed oxygen molecule, from which the superoxide radical is formed [59]. In that case indirect sensitization affect the reaction path of reactive oxygen species (ROS) generation, promoting formation of O_2_^•−^ and decreasing probability of ^•^OH production.

To the best of our knowledge, the procedure for quantitative determination of the photocatalytic conversion of rhodamine B into rhodamine 110 under visible light irradiation has not been described in literature yet. In order to perform the mathematical estimation of this phenomenon, we propose in our study determination of conversion efficiency ($W_{c}$) factor. This parameter is defined by the ratio of the actual number of moles (n_a_) of the substance obtained as a result of chemical transformation, to the theoretical number of moles (n_t_) of this substance that would be obtained as a result of this reaction assuming that all the substrate molecules were converted into product. Conversion efficiency is described by the formula:

(5) $$W_{c}=\frac{n_{a}}{n_{t}}\times 100\%$$

Due to the fact that the chemical transformation of RhB into Rh-110 takes place in the same volume, the number of moles can be converted to molar concentration. After using the Bouguer–Lambert–Beer law, the formula takes the form:

(6) $$W_{c}=\;\frac{A_{Rh-110}\times \varepsilon_{RhB}}{A_{RhB}\times \varepsilon_{Rh-110}}\times 100\%$$

In order to make the appropriate calculations, the molar extinction coefficient (ε) for RhB and Rh-110 was estimated experimentally. It was found ε _RhB_ equals 89 590 cm^−1^ M^−1^ and ε _Rh-110_ equals 70 605 cm^−1^ M^−1^. In the present study conversion coefficient has been calculated using value of A_RhB_ at the peak maximum at the wavelength 554 nm presented by initial RhB solution (with concentration 1 × 10^−5^ M) and A_Rh-110_ at the peak maximum at the wavelength 498 nm measured after 300 min of photocatalytic process.

Determination of conversion efficiency allows also the calculation of removal efficiency ($W_{r}$):

(7) $$W_{r}=100\%-\;W_{c}$$

Removal efficiency describes photocatalytical degradation of RhB molecules, as a result of chromophore destruction.

Conversion efficiency or removal efficiency cannot be considered separately in studies of photocatalytic performance, but should always be analyzed together with the reaction rate constant. Observed processes can be analyzed in terms of the contaminant removal or synthesis of the intended compound.

### 3.4. Analysis of Photocatalytic Properties

**Figure 10:**
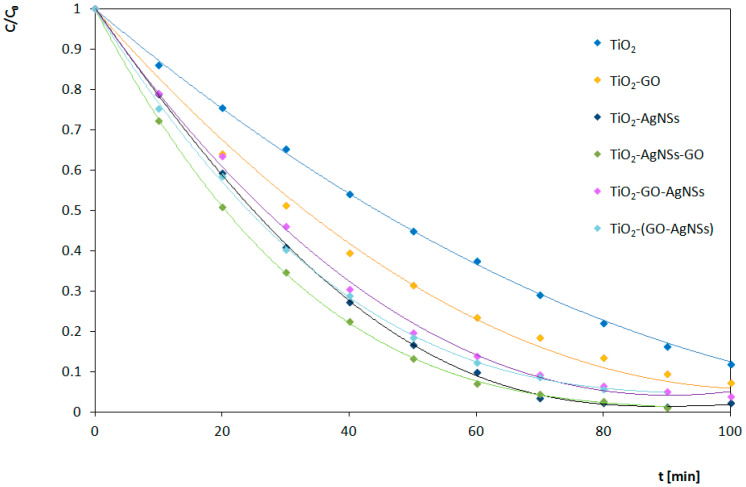
Photocatalytic degradation of RhB under UV irradiation in the presence of prepared photocatalysts.

**Figure 11:**
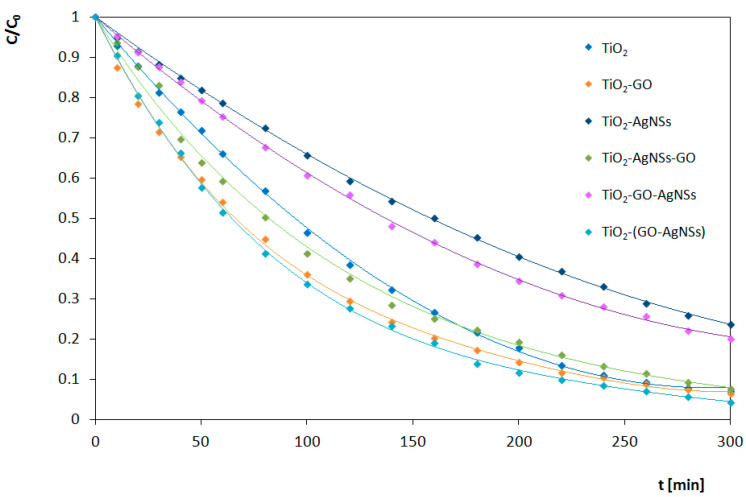
Photocatalytic degradation of RhB under Vis irradiation in the presence of prepared photocatalysts.

Results of the photocatalytic transformations of RhB by different photocatalysts under UV and Vis irradiation are shown in Figure 10 and Figure 11, respectively. Estimated reaction rates and conversion efficiency related to photocatalytic activity of obtained TiO_2_, TiO_2_-GO, TiO_2_-AgNSs, TiO_2_-AgNSs-GO, TiO_2_-GO-AgNSs and TiO_2_-(GO-AgNSs) coatings under UV and Vis irradiation are presented in Table 2. The results clearly indicate that photocatalytic activity depends on the composition and architecture of the coating.

#### 3.4.1. Under UV Irradiation

Involving GO and AgNSs in the photocatalytic system leads to an increase of its activity in the RhB degradation under UV irradiation. In binary systems, the incorporation of AgNSs is more efficient (0.0352 min^−1^) than deposition of GO flakes (0.0256 min^−1^). A high activity of TiO_2_-AgNSs coatings is the result of electrons trapping by the Schottky junction and the possibility of generation of the hydroxyl radicals on the AgNSs surfaces [18].

GO combined with TiO_2_ also acts as an electron sink, resulting in delay in recombination of electron-hole pair [60], but the behavior of this system under UV is more complex. It is known that without access of oxygen GO can be photoreduced on TiO_2_ by excited electron from CB, leading to reconstruction of a conjugated network [61], but with access of air this reaction path is interrupted by a competitive process of oxidation of oxygen moieties localized in a GO structure, which leads to the decomposition of flakes [21,22]. This process depends on the intensity of radiation, and thereby reactive oxygen species production. Moreover, residual oxygen moieties on reduced graphene oxide (RGO) can undergo oxidation by superoxide anion [62]. Herein, the amount of GO in all coatings was identical, which eliminates the impact of the aforementioned phenomena on the comparison of various architectures.

TiO_2_-AgNSs-GO coating shows the best photocatalytic properties in UV light among all the coatings examined in this work. The value of the constant rate of RhB degradation on this coating (0.0405 min^−1^) is almost 2 times higher (exactly 98% higher) the value of the rate constant for unmodified TiO_2_ coating (0.0204 min^−1^). Interestingly, deposition of GO flakes (TiO_2_-GO; 0.0256 min^−1^) leads also to an increase of the reaction rate constant value of unmodified TiO_2_, but only by 25%. However, decoration of TiO_2_ by AgNSs resulting in the TiO_2_-AgNSs photocatalyst (which differs from TiO_2_-AgNSs-GO coating only by the absence of GO flakes on the surface) leads to an increase of this value by 72%. On this basis, it can be concluded that the additive effect increasing the photocatalytic performance of both components (i.e., AgNSs and GO) occurs in the TiO_2_-AgNSs-GO coating. Most likely, this effect is related to the behavior of AgNSs as electron traps, resulting in the suppressing of electron-hole recombination. Furthermore, it is also possible that, due to the fact that GO flakes are located on AgNSs, electrons can be transferred from AgNSs to the GO structure, which causes even greater delay of the recombination process.

The presence of silver and GO appeared also beneficial in other types of architecture. The TiO_2_-(GO-AgNSs) coating (0.0331 min^−1^) differs from the TiO_2_-GO coating (0.0256 min^−1^) only by the presence of AgNSs on the surface of GO flakes. The introduction of AgNSs has further improved the process of photocatalytic decolorization of RhB. The value of the reaction rate constant on the TiO_2_-(GO-AgNSs) coating is 62% higher than the value for the unmodified coating. Compared to TiO_2_-GO, the introduction of AgNSs increased the reaction rate constant by 37%. In the TiO_2_-(GO-AgNSs) coating, the interaction of both components is also revealing, leading to the acceleration of the photocatalytic degradation of RhB in UV light.

In contrast, the TiO_2_-GO-AgNSs (0.0309 min^−1^), which is characterized by areas covered or uncovered with GO flakes, differing in the number and size of AgNSs, also shows an improvement in photocatalytic properties in comparison with TiO_2_-GO. However, the value of the increase of the reaction rate constant is not so high—only 26%. It is worth noting, that for the TiO_2_-(GO-AgNSs), the value of the reaction rate constant compared to TiO_2_-GO was 37% higher and is more than 11% higher than for TiO_2_-GO-AgNSs. Therefore, the AgNSs localized on the TiO_2_ surface not covered with GO flakes may provoke competitive phenomena. The excited electron from TiO_2_ can be transferred both to AgNSs situated on TiO_2_ or to AgNSs connected with GO.

**Figure 12:**
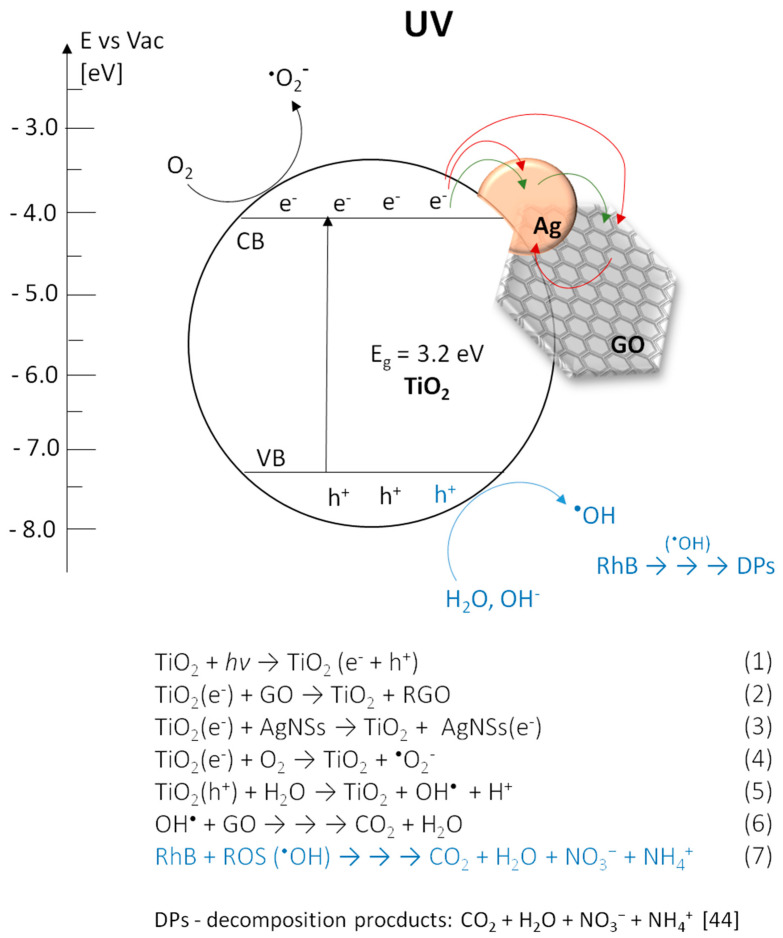
The reaction path [22,44,55,63] and possible photocatalytic mechanism of the three-component systems with different surface architecture occurring for photocatalysis of RhB performed under UV radiation. Green arrows correspond to the flow of electrons in TiO_2_-AgNS-GO composite. Red arrows correspond to the flow of electrons in TiO_2_-(GO-AgNS) and TiO_2_-GO-AgNS composites.

The discussed phenomena occurring in the presence of three-component coatings under the UV radiation are presented in Figure 12.

To summarize the experiments conducted under UV irradiation, it was found that the incorporation of both GO and AgNSs with TiO_2_ leads to enhancement of photocatalytic degradation of RhB. The architecture of the ternary coating intensively affects the photocatalyst activity resulting in significant differences in constant rate values. However, it should be taken into consideration that GO flakes deposited on TiO_2_ surface under that range of irradiation are prone to decompose, which ultimately means that the photocatalysts including them are not stable in that condition. In that case, a strategy to combine TiO_2_ only with AgNSs seem to be more beneficial for the photocatalysts’ applications under UV irradiation.

#### 3.4.2. Under Visible Light (Vis) Irradiation

By contrast with the processes observed in the UV range, under visible light irradiation, the RhB degradation is processing simultaneously with its transformation into Rh-110. As it was mentioned above, the process of N-deethylation is associated with the adsorption process of the dye on the TiO_2_ surface.

It is known that GO is the preferred area for adsorption of RhB molecules by π–π stacking interactions [64]. Moreover, GO that covers the TiO_2_ surface results in competitive adsorption of RhB on GO rather than on semiconductor surface leading to lowering of interactions of the dye with the semiconductor surface via aminoethyl groups [59]. Consequently, this decreases the probability of electron injection from RhB to TiO_2_, which is necessary under Vis irradiation to provide sensitization of TiO_2_. In that case, RhB is not efficiently transformed into Rh-110. Even when the electron transfer occurs, the electron from CB in TiO_2_ can be effectively transported to GO leading to its reduction [61]. However, superoxide radical produced during the RhB N-deethylation process may promote GO oxidation. It is probable that reactive oxygen species and products that arose during GO oxidation can interact with RhB molecules, which promotes chromophore degradation. Therefore, the transformation of RhB to Rh-110 is limited (conversion efficiency valued 53%), but its degradation is accelerated (0.0095 min^−1^).

AgNSs deposited on TiO_2_ cover only 1.1% of the surface, so decreasing the area for adsorption of RhB is negligible. Moreover, AgNSs promote transformation of RhB to Rh-110 by electron trapping, through the Schottky junction, which results in the increase of conversion efficiency to 67% in contrast with bare TiO_2_. Electrons trapped in metallic centers (AgNSs) react with adsorbed oxygen leading to the appearance of superoxide radical ^•^O_2_^¯^ that promotes the transformation of RhB into Rh-110. Therefore, degradation of chromophore is limited, and simultaneously overall process is decelerated. In that case, the value of rate constant for RhB degradation on TiO_2_-AgNS is almost two times lower in comparison with unmodified TiO_2_.

Opposite mechanisms resulting from addition of AgNSs and GO affect the activity of ternary systems simultaneously.

The TiO_2_-(GO-AgNSs) has similar photocatalytic activity to TiO_2_-GO under Vis irradiation. The value of the conventional reaction rate constant for the process carried out on TiO_2_-(GO-AgNSs) equal to 0.0102 min^−1^ is only slightly higher than the rate constant for TiO_2_-GO (0.0095 min^−1^). Equally determined values of the efficiency of the RhB transformation process in Rh-110 are comparable—52% for TiO_2_-(GO-AgNSs) and 53% for TiO_2_-GO. Therefore, it can be concluded that the additional presence of AgNSs in three-component system, has slight effect on the photocatalytic transformation of RhB into Rh-110, but the addition of GO has a dominant effect.

The properties of the TiO_2_-AgNSs-GO coating should be compared with both TiO_2_-GO and TiO_2_-AgNSs. In a two-component system, the addition of GO caused an increase in the value of a constant reaction rate, while the addition of AgNSs caused a decrease. Both of these phenomena were reflected in the properties of the TiO_2_-AgNSs-GO coating. Their opposite effects caused the conventional reaction rate constant for TiO_2_-AgNSs-GO (0.0082 min^−1^) to be slightly lower than the value characterizing unmodified TiO_2_ (0.0088 min^−1^). In addition, the determined yield value of 60% was similar to the value determined for unmodified TiO_2_ (65%). To sum up, the effects from AgNSs and GO eliminate each other.

TiO_2_-GO-AgNSs coating is covered with AgNSs to a greater extent than TiO_2_-AgNSs-GO. Therefore, a decrease in the reaction rate constant value to 0.0053 min^−1^ is visible. This value is similar to the value obtained for the TiO_2_-AgNSs coating (0.0047 min^−1^). The determined efficiency of the RhB transformation process in Rh-110 equal to 55% is much lower than for the TiO_2_-AgNSs coating (67%), but also it is slightly higher than for the TiO_2_-GO coating (53%). Therefore, in the case of TiO_2_-GO-AgNSs coating, the effect of deceleration of the process of RhB photocatalytic transformation resulting from the addition of AgNSs and a decrease in this process efficiency resulting from the addition of GO can be observed.

**Figure 13:**
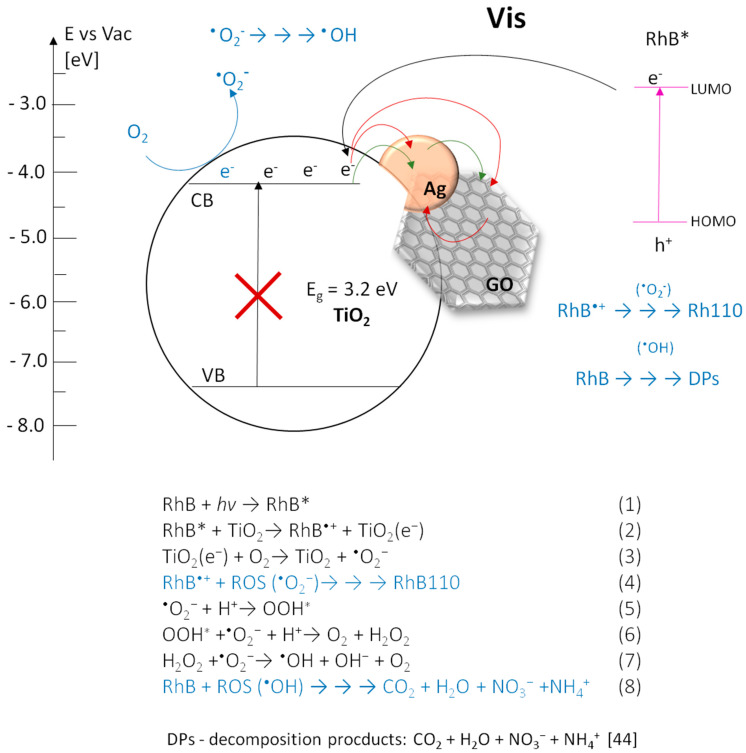
The reaction path [22,44,55,63] and possible photocatalytic mechanism of the three-component systems with different surface architecture occurring for photocatalysis of RhB performed under Vis radiation. Green arrows correspond to the flow of electrons in TiO_2_-AgNS-GO composite. Red arrows correspond to the flow of electrons in TiO_2_-(GO-AgNS) and TiO_2_-GO-AgNS composites.

The discussed phenomena occurring in the presence of three-component coatings under the UV and Vis radiation are presented in Figure 13.

To summarize the Vis experiments, GO exerts a dominant effect on the efficiency of photocatalytic conversion of RhB into Rh-110 in all cases, and also increases the degradation of RhB. By contrast, decoration of TiO_2_ by AgNSs leads to prolongation of the photocatalytic transformation of RhB. However, a small addition of AgNSs located on GO flakes causes a slight acceleration of the process. These observations are in good agreement with the effect of AgNSs electron trapping, elucidated in the mechanism presented by Liu et al. [24].

## 4. Conclusions

This work provides a comprehensive study on ternary TiO_2_ coatings with GO and AgNSs and unravels which components affect the photocatalytic properties of the final materials. Received data indicate that the influence of both modifications is significant and differ under particular range of light. The impact of the AgNSs contribution is rather complex. Decoration with AgNSs leads to higher reaction rate constants in the UV radiation range, whereas under Vis irradiation it elongates decomposition of RhB improving at the same time RhB conversion efficiency. By contrast, the presence of GO increases the decomposition rate of RhB in both selected ranges of radiation. Under Vis illumination, the impact of GO in ternary systems is usually dominant. A synergistic effect of both components is not observed due to favoritism of opposite processes under the applied conditions. Only in the case of TiO_2_-GO-AgNSs architecture under UV irradiation the synergism of both modifications influencing the degradation of RhB was observed. Hence, this work indicates that architecture of photocatalyst surface has huge impact on its photocatalytic activity and this impact can differ in selected range of radiation.

Moreover, phenomena which occur during photochemical processes involving RhB were discussed. It was found that under UV irradiation RhB undergoes only photodegradation, while in the visible light range its transformation into rhodamine 110 (Rh-110) is preferred. This can be explained by the fact that UV irradiation causes the appearance of ^•^OH radicals that decompose RhB by the oxidation path. In the case of the Vis range, the formation of superoxide radical ^•^O_2_^−^, that can be responsible for promotion of chemical transformation of RhB to Rh-110, is favored. Further conversion of ^•^O_2_^−^ to ^•^OH may also occur which leads to RhB mineralization [55]. However, transformation into Rh-110 prevails. A simple and novel method for the estimation of the photocatalytic activity in terms of transformation of RhB into Rh-110 was proposed.

Most of the actual studies focus on examining the effectiveness of photocatalytic systems in artificial solar light conditions, due to striving to conduct the process with the use of our natural energy source. In the presented work, it was indicated that the processes occurring in the selected range of light can have different reaction paths, which can interrupt each other under solar light irradiation. That phenomenon should be taken into consideration when designing photocatalysts for tailored applications.

## Figures and Tables

**Table 1:** Average size, maximal diameter and surface density of obtained AgNSs on specific areas of prepared photocatalysts.

Type of Coating	Synthesis Conditions	Area	Average Size [nm]	Maximal Diameter [nm]	Surface Density [AgNSs/µm^2^]
TiO_2_-AgNSs	under UV	on TiO_2_	18 ± 3	37	55
TiO_2_-AgNSs-GO	under UV	on TiO_2_	18 ± 3	37	55
		under GO	18 ± 3	37	55
TiO_2_-GO-AgNSs	under UV	on TiO_2_	19 ± 3	39	20
		on GO	26 ± 4	66	35
TiO_2_-(GO-AgNSs)	darkroom	on TiO_2_	-	-	-
		on GO	19 ± 3	53	3

**Table 2:** Reaction rate constants estimated for coatings with defined architecture, established for UV and Vis radiation and conversion efficiency of RhB transformation calculated for process under Vis irradiation.

Type of Coating	UV	Vis	
	k’ [min^−1^] *	k’ [min^−1^] *	W_c_ [%]
TiO_2_	0.0204	0.0088	65
TiO_2_-GO	0.0256	0.0095	53
TiO_2_-AgNSs	0.0352	0.0047	67
TiO_2_-AgNSs-GO	0.0405	0.0082	60
TiO_2_-GO-AgNSs	0.0309	0.0053	55
TiO_2_-(GO-AgNSs)	0.0331	0.0102	52

*—The error (standard deviation) of reaction rate constant is lower that 5%.
